# A Machine Learning Approach for Predicting Capsular Contracture after Postmastectomy Radiotherapy in Breast Cancer Patients

**DOI:** 10.3390/healthcare11071042

**Published:** 2023-04-05

**Authors:** Domenica Antonia Bavaro, Annarita Fanizzi, Serena Iacovelli, Samantha Bove, Maria Colomba Comes, Cristian Cristofaro, Daniela Cutrignelli, Valerio De Santis, Annalisa Nardone, Fulvia Lagattolla, Alessandro Rizzo, Cosmo Maurizio Ressa, Raffaella Massafra

**Affiliations:** I.R.C.C.S. Istituto Tumori “Giovanni Paolo II”, Viale Orazio Flacco 65, 70124 Bari, Italy

**Keywords:** immediate reconstruction, radiotherapy, capsular contracture, machine learning, prothesis, expander

## Abstract

In recent years, immediate breast reconstruction after mastectomy surgery has steadily increased in the treatment pathway of breast cancer (BC) patients due to its potential impact on both the morpho-functional and aesthetic type of the breast and the quality of life. Although recent studies have demonstrated how recent radiotherapy techniques have allowed a reduction of adverse events related to breast reconstruction, capsular contracture (CC) remains the main complication after post-mastectomy radio-therapy (PMRT). In this study, we evaluated the association of the occurrence of CC with some clinical, histological and therapeutic parameters related to BC patients. We firstly performed bivariate statistical tests and we then evaluated the prognostic predictive power of the collected data by using machine learning techniques. Out of a sample of 59 patients referred to our institute, 28 patients (i.e., 47%) showed contracture after PMRT. As a result, only estrogen receptor status (ER) and molecular subtypes were significantly associated with the occurrence of CC after PMRT. Different machine learning models were trained on a subset of clinical features selected by a feature importance approach. Experimental results have shown that collected features have a non-negligible predictive power. The extreme gradient boosting classifier achieved an area under the curve (AUC) value of 68% and accuracy, sensitivity, and specificity values of 68%, 64%, and 74%, respectively. Such a support tool, after further suitable optimization and validation, would allow clinicians to identify the best therapeutic strategy and reconstructive timing.

## 1. Introduction

Breast cancer represents the most common cancer disease in women and also is the leading cause of cancer-related death [1]. According to data from the Italian Association of Cancer Registry (AIRTUM), an estimated 55,000 new cases of malignant breast cancer were diagnosed in our country in the last year, accounting for about 30% of all new cancer diagnoses [2].

In recent years, immediate breast reconstruction with prosthesis or expander, after mastectomy or conservative surgery, has steadily increased [3], being valuable in the treatment of breast cancer, as well as in the plastic surgery practice, due to its potential impact on both the morpho-functional and aesthetic type of the breast and the relative quality of life [4,5].

Given the high incidence of the disease and, consequently, the large number of breast reconstructions, both surgical results and quality of life for patients undergoing surgery and radiation therapy must be optimized [6].

Based on clinical, pathological, and tumor characteristics, some patients need to undergo several cycles of radiotherapy. In recent years, the indication for radiation therapy is increasingly recommended for patients with locally advanced breast cancer [7,8].

The latest developments in the field of reconstructive plastic surgery are coupled by constantly evolving radiotherapy fractionation methods [9]. Radiation therapy can affect outcomes after breast reconstruction, depending on the type of RT technique (three-dimensional conformal RT [3D CRT] vs. intensity-modulated radiation therapy [IMRT]), timing of RT (proceeding vs. after reconstruction), and size of fractionation (hypofractionation vs. conventional fractionation) [10]. To date, studies are ongoing in the context of conventional and hypofractionated radiotherapy fractionation to understand the association between radiation dose and complication rate in patients undergoing breast reconstruction [11,12].

However, neither surgical nor radiotherapy procedures are free of complications, although recent studies report that these, along with the cosmetic results of prosthetic reconstruction under post mastectomy radiation therapy (PMRT), have reached more acceptable levels with respect to the past [13,14]. 

**Figure 1:**
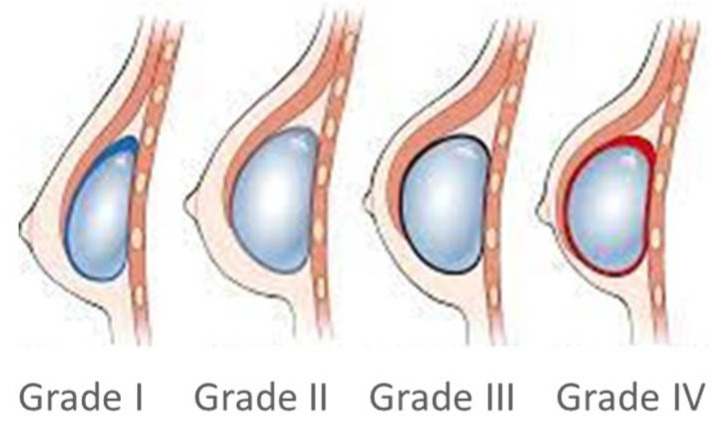
Capsular contracture occurs when either the normal healing process fails or when a pathological change, caused by tissue trauma or an exogenous trigger, started and can be of differing severity according to the Baker classification.

Capsular contracture is the main complication after breast implant surgery [15]. Capsular contracture occurs when either the normal healing process fails or when a pathological change caused by tissue trauma or an exogenous trigger are started. This pathological condition is named Baker’s contracture, from grade I, absent, to IV, severe (Figure 1).

Most studies have shown a higher frequency of contracture for smooth implants than for those with textured surfaces [16]. Currently, the exact cause of contracture is still unclear; it has been hypothesized that immunobiological factors (i.e., biological and bacterial factors) and various risk factors play a key role in its development.

Although capsular contracture is the main complication in prosthetic implants, there is no pathognomonic radiological sign of contracture, and the diagnosis is mainly clinical: there are different clinical pictures, sometimes vague, which do not always allow an early and easy diagnosis, and the preparation of a diagnostic prediction algorithm could help clinicians in identifying subjects at risk.

Several studies have been proposed in the literature with the aim of evaluating the factors associated with the onset of capsular contracture following radiotherapy. However, the efforts in defining a predictive model of the same using artificial intelligence techniques are currently limited [17].

In this paper, we proposed a preliminary model to predict the complications of radiotherapy after mastectomy trained on with the purpose of undertaking the best treatment course and reconstructive timing for each patient, that is, to identify patients with a higher predicted risk of postoperative reconstructive complications. The aim of this study is to evaluate the predictive power of clinical characteristics commonly collected in clinical practice by means of a machine learning model well known with regards to the state-of-the-art. Specifically, we analyzed complications of radiotherapy in patients with prosthesis or expander who underwent mastectomy surgery in our institute from 2016 to 2021. To this aim, we developed a retrospective database containing clinical and surgical features of 59 breast cancer patients.

The analyzed features included modifiable and non-modifiable risk factors, such as smoking and age, allergies and comorbidities, and tumor characteristics. Surgical characteristics refer to the type of prosthesis or expander inserted, volume of expander, lymphadenectomy, and, of course, date of radiotherapy and any complications.

## 2. Materials and Methods

### 2.1. Experimental Data

**Figure 2:**
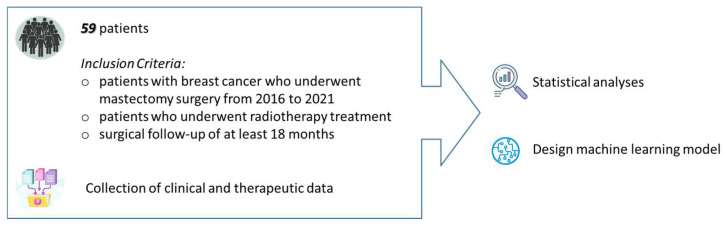
Data analytics framework.

This study included breast cancer patients who underwent reconstructive surgery and radiotherapy from 2016 to 2021 at Istituto Tumori “Giovanni Paolo II” in Bari (Italy). All patients who underwent radiotherapy after mastectomy at our Institute were recruited, for whom who also had surgical follow-up of at least 18 months. The clinical and therapeutic characteristics extracted from our information systems were collected (Figure 2).

Specifically, we collected 59 patients who have received radiotherapy after the implantation of prosthesis or expander. No matrices were used in the reconstructions. All patients were treated with the three-dimensional conformal technique (3DRT) and received a total dose of 50 Gy with a daily fractionation of 2 Gy in 25 fractions. The technique involves tangent bundles, with 95% of the prescribed dose covering at least 95% of the target.

We anonymized all data before proceeding with the analysis, and we did not require written consent for this retrospective study with minimal risk. The study was conducted according to the guidelines of the Declaration of Helsinki and approved by the scientific board of our institute.

For each patient, we collected the following clinical, therapeutic, and surgical characteristics: age, menopausal status (premenopausal/postmenopausal), estrogen receptor status (ER, Neg/Pos), progesterone receptor status (PgR, Neg/Pos), cellular marker for proliferation status (ki67, Neg/Pos with cut-off 20%), human epidermal growth factor receptor-2 status (HER2, Neg/Pos), histological grade (G, Elston–Ellis scale: 1, 2, 3), tumor size (T1, T2, T3), lymph-node status (N0, N1, N2, N3), histological subtype (ductal, other special types), molecular subtype (HER2 positive, luminal A, luminal B, and triple negative), neoadjuvant chemotherapy (yes/no), lymphadenectomy (yes/no), type of surgery (expander or prosthesis), and months between reconstruction time and start of radiotherapy.

We also considered information about the size of the implant. Three size classes have been identified, that is, up to 300cc, from 300cc to 450cc, and greater than 450cc, where “cc” stands for “cubic centimeter”.

Moreover, we collected the occurrence of contracture events after radiotherapy.

### 2.2. Statistical Analyses

In order to study the statistical association between clinical characteristics with the contracture on prosthesis or expander after radiotherapy, we used the chi-square test for categorical variables and Mann-Whitney’s test for continuous variables. 

The chi-square test is a non-parametric test, which is used for testing independence [18]. It can be employed with categorical variables in order to study if the association between these variables is statistically significant. 

The null hypothesis consists in the independence between the two variables, while the alternative hypothesis considers the dependence between the two variables. 

Mann-Whitney’s test is a non-parametric test that is used to study the outcomes comparison between two independent groups. The null hypothesis considers the equality of the two populations, while the alternative hypothesis is about the unequal populations [19].

We considered the result as statistically significant when the test returned a *p*-value less than 0.05.

### 2.3. Classification Model

In order to predict the contracture event, we trained a classification model on 15 features, which were common to the two methods (expander and prosthesis), i.e., age at diagnosis, menopausal status, estrogen receptor status (ER), progesterone receptor status (PgR), ki67 proliferation status, human epidermal growth factor receptor-2 status (HER2), histological grade, tumor size, lymph nodes, histological subtype, molecular subtype, neoadjuvant chemotherapy, lymphadenectomy, type of surgery, and months between reconstruction and radiotherapy. Missing values were estimated through Miss Forest, a tool provided by Random Forest, before analyzing data.

We compared three standard classification algorithms, such as Random Forest (RF), eXtreme Gradient Boosting (XGBoost), and Support Vector Machines (SVM).

Random Forest is a supervised learning algorithm based on a set of decision trees, which are usually trained by a method named “bagging”, according to which the overall result is augmented by a combination of learning patterns. [20,21,22].

It reduces the risk of overfitting, which may be caused by all samples in the training data. If the number of decision trees is robust, the overall variance and prediction error will be reduced, so the classifier will not overfit the model. The RF classification algorithm basically depends on two parameters, such as the number of trees and the number of features to choose in each node split. In this work, we have adopted a standard configuration with 100 trees and 20 features [as described in Breiman (24)] randomly selected at each subdivision. Additionally, to control the risk of overfitting, we set a small number of observations per tree leaf, such as 5.

XGBoost stands for “Extreme Gradient Boosting” [23], and it is a decision tree ensemble learning algorithm similar to random forest, which combines multiple machine learning algorithms to obtain a better model. The difference is how the trees are built and combined. XGBoost is a boosting technique that sequentially creates decision trees, where each tree improves upon the mistakes of the previous one by exploiting a gradient descent optimization algorithm for minimizing the loss function. Since the Gradient Boosting algorithm could overfit a training dataset, regularization methods are implemented for improving performance. XGBoost has parallel and distributed processing, which allows it to be faster than other algorithms. The goal is to provide a library that is scalable, portable, and accurate, with a lot of focus on optimization systems and machine learning principles.

The Support Vector Machine (SVM) classification algorithm [24] estimates a hyperplane separating points in a high dimensional space, so that the examples of the categories are divided by a clear gap. New examples are then mapped into that same space and are predicted to belong to a category based on the side of the gap on which they fall. In this work, we used a linear kernel. 

For each of the classification algorithms, the default parameters were used. More complicated architectures did not give any significant classification improvements.

The three above-described classification algorithms were implemented after performing a feature selection procedure with Random Forest and determining feature importance through “Mean Decrease Accuracy”, which measures the accuracy lost by the model in the exclusion of each variable. Only the features with an index of importance higher than the average importance of the initial set of features were then selected. Specifically, the feature selection procedure is nested in the cross-validation.

The models were evaluated on 10 rounds of a 10-fold cross-validation, and the classification performances were evaluated in terms of ROC AUC value and accuracy, sensitivity, and specificity, which was calculated according to an optimal threshold obtained from Youden’s index on the ROC curves [25].

All the analyses were performed by using the MATLAB R2022a (MathWorks, Inc., Natick, MA, USA) software.

## 3. Results

### 3.1. Statistical Analysis Results

Table 1 shows the clinical and treatment characteristics of the analyzed patients. Patients who received radiotherapy on prosthesis or expander were 59, with a median age of 47 years.

These have been divided into two groups according to the occurrence or non-occurrence of contracture following radiotherapy. Specifically, 28 patients had contracture, representing 47% of the sample, while the remaining patients did not have contracture. The sample was composed of 47% of patients who did radiotherapy on expander, while 53% of patients did radiotherapy on prosthesis. Observed contractures were mostly moderate or severe, i.e., grades 3 and 4, according to Baker’s classification. Specifically, 7% of the overall capsular contracture were grade 2, 76% were grade 3, and 17% were grade 4.

**Figure 3:**
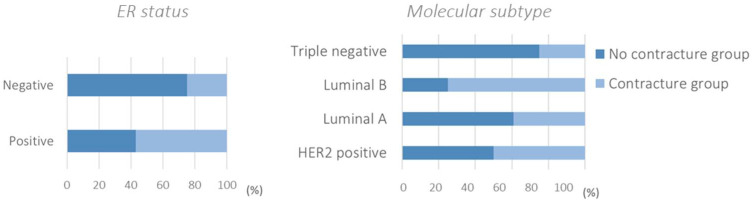
ER and molecular subtypes were statistically associated with capsular contracture after postmastectomy radiotherapy (*p*-value < 0.10).

The significance of the variables compared with the onset of contracture was assessed using two statistical tests, in particular the chi-square test for categorical variables and the Mann-Whitney test for continuous variables, such as age and months between reconstruction and RT. The statistically significant variable was ER, which had a *p*-value less than 0.05. About 57% of ER positive patients and 25% of ER negative patients had contracture (Figure 3). We underline also the molecular subtype, which showed a significance just above the 0.05 cut-off considered. Specifically, it emerged among the luminal A molecular subtypes, and approximately 61% had no contracture, while among the luminal B molecular subtypes, approximately 75% had contracture. Considering the triple negative patients, 75% had no contracture.

### 3.2. Performance Evaluation Results

**Figure 4:**
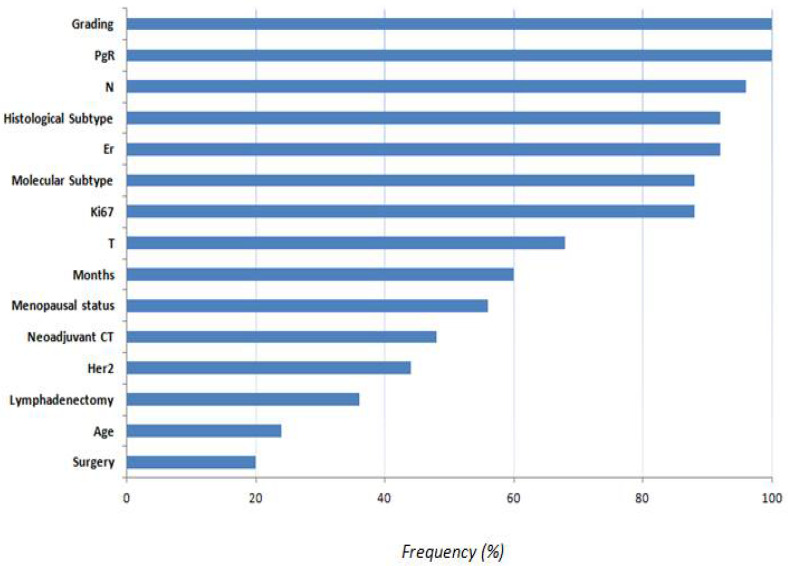
Frequency of the selected features.

We identified the most important features as those selected by implementing Random Forest “mean decrease accuracy” technique. The average number of features selected in each round of cross-validation was seven features. Figure 4 shows the frequency of the selected features on a 10 ten-fold cross-validation scheme. Features with lower selection frequency were age, lymphadenectomy, and surgery. This means that the type of surgery, that is expander or prosthesis, is not a discriminated factor in predicting contracture after receiving radiotherapy. Conversely, months between prosthesis or expander reconstruction and RT had a frequency equal to 60%.

The following tables summarise the performances metrics evaluated within a cross-validation scheme in terms of median value and interquartile range (1th and 3th quantiles). Experimental results considering patients with expander or prosthesis (Table 2) have shown that XGB performed better than RF and SVM in terms of AUC, although the other evaluation metrics were comparable among all the classifiers. Specifically, the XGB classifier showed an AUC value of 68% and accuracy, sensitivity, and specificity values of 68%, 64%, and 74%, respectively. SVM and XGB classifiers reached results, which were more balanced if compared to the RF classifier.

Specifically, the accuracies recorded by the three classifiers, i.e., RF, SVM, and XGB, were 50%, 54%, and 64%, respectively. For patients with prostheses, the classification performances rose to 71%, 74%, and 71%, thus showing how the collected characteristics seem to better predict a PMRT contracture event for this type of implantation.

## 4. Discussion and Conclusions

Breast reconstruction with prosthesis or expander after mastectomy is a fundamental step in breast cancer treatment, as well as in plastic surgery practice. Thus, clinicians choose which patients are eligible for radiotherapy cycles according to their clinical, pathological, and tumor characteristics. Radiotherapy is a localized and non-invasive treatment, which allows us to kill cancer cells and reduce tumors through the use of high-energy radiation [26]. RT in breast cancer has the task of reducing the chance of recurrence and improving breast cancer survival rate. RT is essential, but it can cause the onset of different post reconstructive surgery complications, including contractures [27].

Most of the research works in the state-of-the-art aim at analyzing, in a univariate manner, possible factors associated with capsular contracture after post-radiotherapy in mastectomised patients, focusing on the comparison between different reconstruction methods or therapeutic approaches. In a previous meta-analysis study [28] made by Fengzhou Du and colleagues, the authors evaluated the results of the DTI ("direct-to-implant”) and TEI (“tissue expander after implant”) procedures followed by PMRT (“post-mastectomy radiotherapy”). They found that there was not a difference in terms of rate of capsular contracture between implants and tissue expanders, and, in fact, patients who received PMRT, with permanent implant or tissue expander, showed the same risk contracture.

Another study [29] divided patients between adjuvant or neoadjuvant chemotherapy, analysing adverse events by rates, risk factors, therapy, and duration of radiation therapy. Their results showed the connection between neoadjuvant chemotherapy and adverse events in patients undergoing immediate breast reconstruction.

On the other hand, there is a lack of works aiming at the definition of support systems to predict capsular contracture by using artificial intelligence techniques, whose potential is now consolidated in various clinical settings and endpoints of interest [17]. The aim of this preliminary work is to analyse the association between clinical characteristics with the risk of contracture on a set of patients referred to our institute in order to design a preliminary machine learning model on clinical features, and the idea behind this preliminary work is to evaluate how much an artificial intelligence model trained solely on clinical variables commonly collected in clinical practice can predict the onset of post-radiotherapy contractures in mastectomised patients.

Our experimental results related to real-life data show that there is a significant association between ER with contracture. In particular, the likelihood of contracture is high for ER positive patients. Moreover, regarding the molecular subtype, the difference appears to be in the luminal B group, with a *p*-value just above the 0.05 significance level. However, in contrast with our assumptions, there is no significant association of contracture with the type of reconstruction and not even with the time between reconstruction and RT.

After collecting all data potentially predictive of contracture in patients undergoing radiotherapy on expander or prosthesis, we have selected a subset of significant features and trained a machine learning prediction model. Specifically, some standard machine learning models were trained and validated according to a cross-validation procedure.

The features that have shown a high prognostic power were PgR and histological grading, as well as ER, lymph node status, histological subtype, ki67, and molecular subtype. To the contrary, the type of reconstructive surgery and age were the features with the lowest predictive value. The classification performances have demonstrated that the characteristics collected have a non-negligible predictive power, although they are not sufficient for the clinical applicability of the tool. The XGB classifier showed an AUC value of 68% and accuracy, sensitivity, and specificity values of 68%, 64%, and 74%, respectively. Our study represents a preliminary proposal for the development of a tool that can support clinicians in predicting contractures after PMRT. What emerges, and it is important to emphasize to an audience of biomedical data scientists and clinicians, is that the informative power contained in the characteristics considered in this study for the prediction of complications after radiotherapy in mastectomised patients is not negligible. However, the experimental results suggest that there are other personal characteristics of the patient, such as the texture characteristics of the breast, which go beyond the therapeutic path that must necessarily be shaped and integrated into the model.

Recently, Naoum et al. [17] proposed a nomogram to predict the risk of breast reconstruction complications with or without postmastectomy radiation therapy by using machine learning models. The strength of the proposed study is the sample size and inclusion of 56 predictors collected from each patient, including demographics, tumor biology, and various treatment details. Nonetheless, the performance of the implemented logistics model reaches an AUC value of 75%. The proposed model was trained on a highly heterogeneous set of patients with a follow-up of 1 to 18 years, and it did not take into account the changes in surgical techniques throughout the years. 

Although our study is based on a relatively small population, it is a homogeneous case study for the radiotherapy treatment used. However, the study has some limitations related to the number of cases. Indeed, the proposed model needs to be validated and optimized on a large sample, with respect to which other clinical and therapeutic characteristics not considered in this preliminary study can be evaluated, as well as a more accurate evaluation of hyperparameter of the proposal models. In addition, state-of-the-art machine learning models have been used, although recently, innovative approaches have been proposed, which see the use of deep learning techniques also on structured data, such as clinical data [30,31]. Other interesting information could be to evaluate the results in patients with genetic mutations with prosthetic reconstruction and in patients diagnosed with periprosthetic large T-cell lymphoma (Bi-ALCL), where prosthetic contracture is included in the differential diagnosis [32,33].

In future works, by collecting a larger data sample, we will carry out a more accurate tuning phase of the proposed models and also evaluate innovative deep learning models, which could be developed in relation to our clinical data. Moreover, we will integrate radiomic characteristics extracted from diagnostic images with therapeutic and clinical information to define a more accurate personalised model for predicting post-radiotherapycontractures. The definition of an artificial intelligence-based support tool, after suitable optimization and validation, would allow the clinician to identify the best therapeutic strategy and reconstructive timing. Such a personalized medicine model paves the way to reduce complications of radiotherapy, impacting both healthcare cost systems and the quality of women’s vision.

## Figures and Tables

**Table 1:** Clinical and treatment characteristics.

Variable		Total	No Contracture Group	Contracture Group	*p*
Number of Patients		59	31 (53%)	28 (47%)	
Age	Median (IQR)	47 (43–52.5)	47 (43–54)	47.5 (43.5–52)	0.693
Menopausal Status					0.482
	Pre	33	16 (48%)	17 (52%)	
	Post	26	15 (58%)	11 (42%)	
ER					0.05
	Positive	42	18 (43%)	24 (57%)	
	Negative	12	9 (75%)	3 (25%)	
	Missing	5	4 (80%)	1 (20%)	
PgR					0.129
	Positive	39	17 (44%)	22 (56%)	
	Negative	15	10 (67%)	5 (33%)	
	Missing	5	4 (80%)	1 (20%)	
Ki67					0.402
	Positive	33	15 (45%)	18 (55%)	
	Negative	21	12 (57%)	9 (43%)	
	Missing	5	4 (80%)	1 (20%)	
HER2					1.0000
	Positive	12	6 (50%)	6 (50%)	
	Negative	42	21 (50%)	21 (50%)	
	Missing	5	4 (80%)	1 (20%)	
Grading					0.293
	I	2	1 (50%)	1 (50%)	
	II	21	13 (62%)	8 (38%)	
	III	28	11 (39%)	17 (61%)	
	Missing	8	6 (75%)	2 (25%)	
T					0.571
	1	19	11 (58%)	8 (42%)	
	2	24	11 (46%)	13 (54%)	
	3	8	3 (37.5%)	5 (62.5%)	
	Missing	8	6 (75%)	2 (25%)	
N					0.219
	0	9	5 (56%)	4 (44%)	
	1	14	10 (71%)	4 (29%)	
	2	15	6 (40%)	9 (60%)	
	3	14	5 (36%)	9 (64%)	
	Missing	7	5 (71%)	2 (29%)	
Histological subtype					0.482
	Ductal	42	22 (52%)	20 (48%)	
	Other	10	4 (40%)	6 (60%)	
	Missing	7	5 (71%)	2 (29%)	
Molecular subtype					0.076
	HER2 positive	12	6 (50%)	6 (50%)	
	Luminal A	18	11 (61%)	7 (39%)	
	Luminal B	16	4 (25%)	12 (75%)	
	Triple negative	8	6 (75%)	2 (25%)	
	Missing	5	4 (80%)	1 (20%)	
Neoadjuvant chemotherapy					0.0599
	Yes	21	12 (57%)	9 (43%)	
	No	38	19 (50%)	19 (50%)	
Lymphadenectomy					0.449
	Yes	51	25 (49%)	26 (51%)	
	No	4	3 (75%)	1 (25%)	
	Missing	4	3 (75%)	1 (25%)	
Expander or Prosthesis					0.232
	Expander	28	17 (61%)	11 (39%)	
	Prosthesis	31	14 (45%)	17 (55%)	
Size expander					0.285
	≤ 300 cc	5	4 (80%)	1 (20%)	
	300–450 cc	20	11 (55%)	9 (45%)	
	> 450 cc	3	2 (67%)	1 (33%)	
Size prosthesis					0.092
	≤ 300 cc	5	4 (80%)	1 (20%)	
	300–450 cc	12	3 (25%)	9 (75%)	
	> 450 cc	13	7 (54%)	6 (46%)	
	Missing	1			
Months between Reconstruction and RT	Median (IQR)	3 (2–5)	4 (2–7)	3 (1.5–4.5)	0.274

**Table 2:** Metric performances considering patients with expander or prosthesis. The table shows the median value and the first and third quartiles (in brackets) of the performance metrics calculated over the 10 rounds of the 10-fold cross-validation.

Classifier	AUC (%)	Acc (%)	Sensitivity (%)	Specificity (%)
RF	65 (60–65)	64 (61–64)	82 (50–86)	48 (39–74)
SVM	66 (54–68)	66 (59–68)	64 (64–79)	65 (58–68)
XGB	68 (59–66)	68 (61–68)	64 (54–75)	74 (52–81)

## Data Availability

Data from this study are available upon request, since data contain potentially sensitive information. The data request may be sent to the scientific direction (e-mail: dirscientifica@oncologico.bari.it).

## References

[1] van Mourik A.M., Elkhuizen P.H., Minkema D., Duppen J.C., van Vliet-Vroegindeweij C., Dutch Young Boost Study Group (2010). Multiinstitutional study on target volume delineation variation in breast radiotherapy in the presence of guidelines. Radiother. Oncol..

[2] https://www.salute.gov.it/imgs/C_17_notizie_5681_0_file.pdf.

[3] Olsen M.A., Nickel K.B., Fox I.K., Margenthaler J.A., Wallace A.E., Fraser V. (2017). Comparison of wound complications after immediate, delayed, and secondary breast reconstruction procedures. JAMA Surg..

[4] Stuart S.R., Munhoz A.M., Chaves C.L.G., Montag E., Cordiero T.C.S., Fuzisaki T.T., Marta G.N., Carvalho H.A. (2021). Complications after breast reconstruction with alloplastic material in breast cancer patients submitted or not to post mastectomy radiotherapy. Rep. Pract. Oncol. Radiother..

[5] Al-Ghazal S.K., Sully L., Fallowfield L., Blamey R.W. (2000). The psychological impact of immediate rather than delayed breast reconstruction. EJSO.

[6] Checketts J.X., Gordon J., Crawford J.H., Adams H., Duckett L., Vassar B.M. (2018). Is the Right Research Being Conducted to Advance Knowledge about Breast Reconstruction? An Analysis of the Research Pipeline. Plast. Reconstr. Surg..

[7] Carlson R.W., Allred D.C., Anderson B.O., Burstein H.J., Carter W.B., Edge S.B., Erban J.K., Farrar W.B., Goldstein L.J., Gradishar W.J. (2009). Pannello delle linee guida per la pratica clinica del cancro al seno NCC N. Cancro al seno. Linee guida per la pratica clinica in oncologia. J. Natl. Compr. Cancer Netw..

[8] Razdan S.N., Cordeiro P.G., Albornoz C.R., Disa J.J., Panchal H.J., Ho A.Y., Momoh A.O., Matros E. (2017). National Breast Reconstruction Utilization in the Setting of Postmastectomy Radiotherapy. J. Reconstr. Microsurg..

[9] De Rose F., Fogliata A., Franceschini D., Cozzi S., Iftode C., Stravato A., Tomatis S., Masci G., Torrisi R., Testori A. (2019). Postmastectomy radiation therapy using VMAT technique for breast cancer patients with expander reconstruction. Med. Oncol..

[10] Ho A.Y., Hu Z.I., Mehrara B.J., Wilkins E.G. (2017). Radiotherapy in the setting of breast reconstruction: Types, techniques, and timing. Lancet Oncol..

[11] Chang J.S., Song S.Y., Oh J.H., Lew D.H., Roh T.S., Kim S.Y., Keum K.C., Lee D.W., Kim Y.B. (2019). Influence of Radiation Dose to Reconstructed Breast Following Mastectomy on Complication in Breast Cancer Patients Undergoing Two-Stage Prosthetic Breast Reconstruction. Frontiers.

[12] Kim D.Y., Park E., Heo C.Y., Jin U.S., Kim E.K., Han W., Shin K.H., Kim I.A. (2021). Hypofractionated versus conventional fractionated radiotherapy for breast cancer in patients with reconstructed breast: Toxicity analysis. Breast.

[13] Ascherman J.A., Hanasono M.M., Newman M.I., Hughes D.B. (2006). Implant Reconstruction in Breast Cancer Patients Treated with Radiation Therapy. Plast. Reconstr. Surg..

[14] Park T.H., Chung S.W., Song S.Y., Lew D.H., Roh T.S., Lee D.W. (2018). The use of acellular dermal matrix in immediate two-stage prosthetic breast reconstruction provides protection from postmastectomy radiation therapy: A clinicopathologic perspective. J. Mater. Sci. Mater. Med..

[15] Spear S.L., Baker J.L. (2015). Classification of capsular contracture after prosthetic breast reconstruction. Plast. Reconstr. Surg..

[16] Malata C.M., Feldberg L., Coleman D.J. (1997). Textured or smooth implants for breast augmentation? Three year follow-up of a prospective randomised controlled trial. Br. J. Plast Surg..

[17] Naoum G.E., Ho A.Y., Shui A., Salama L., Goldberg S., Arafat W., Winograd J., Colwell A., Smith B.L., Taghian A.G. (2021). Risk of developing breast reconstruction complications: A machine-learning nomogram for individualized risk estimation with and without postmastectomy radiation therapy. Plast. Reconstr. Surg..

[18] Kim H.Y. (2017). Statistical notes for clinical researchers: Chi-squared test and Fisher’s exact test. Restor. Dent. Endod..

[19] Fay M.P., Malinovsky Y. (2018). Confidence intervals of the Mann-Whitney parameter that are compatible with the Wilcoxon-Mann-Whitney test. Stat. Med..

[20] Schonlau M., Zou R.Y. (2020). The random forest algorithm for statistical learning. Stata J..

[21] Sruthi E.R. (2022). Understanding Random Forest. https://towardsdatascience.com/understanding-random-forest-58381e0602d2.

[22] Rigatti S.J. (2017). Random forest. J. Insur. Med..

[23] Brownlee J. A Gentle Introduction to XGBoost for Applied Machine Learning. XGBoost 2016. https://machinelearningmastery.com/gentle-introduction-xgboost-applied-machine-learning/.

[24] Ed-Daoudy A., Maalmi K. (2020). Breast cancer classification with reduced feature set using association rules and support vector machine. Netw. Model. Anal. Health Inform. Bioinform..

[25] Youden W.J. (1950). Index for rating diagnostic tests. Cancer.

[26] DePolo J. Radiation Therapy. Breastcancer.org.

[27] Chung S.Y., Chang J.S., Shin K.H., Kim J.H., Park W., Kim H., Kim K., Lee I.J., Yoon W.S., Cha J. (2021). Impact of radiation dose on complications among women with breast cancer who underwent breast reconstruction and post-mastectomy radiotherapy: A multi-institutional validation study. Breast.

[28] Kanda M.H., da Costa Vieira R.A., Lima J.P.S.N., Paiva C.E., De Araujo R.L.C. (2020). Late locoregional complications associated with adjuvant radiotherapy in the treatment of breast cancer: Systematic review and meta-analysis. J. Surg. Oncol..

[29] Adachi Y., Okumura S., Sawaki M., Hattori M., Yoshimura A., Gondo N., Kotani H., Iwase M., Kataoka A., Sugino K. (2020). Effects of neoadjuvant chemotherapy on operative adverse events and chemotherapy and radiotherapy in patients undergoing immediate breast reconstruction. Breast Cancer.

[30] Dahouda M.K., Joe I. (2021). A deep-learned embedding technique for categorical features encoding. IEEE Access.

[31] Sharma A., Vans E., Shigemizu D., Boroevich K.A., Tsunoda T. (2019). DeepInsight: A methodology to transform a non-image data to an image for convolution neural network architecture. Sci. Rep..

[32] Ibrahim A.E., Atiyeh B.S., Sarhane K.A., Clemens M. (2016). Breast Implant Associated Anaplastic Large Cell Lymphoma (ALCL): Current Recommendations on Diagnosis and Treatment Strategies. Aesthetic Surg. J..

[33] Pieszko K., Kuczyński M., Murawa D. (2018). Breast implant-associated anaplastic large cell lymphoma (BIA-ALCL)—How to diagnose and treat?. Nowotwory J. Oncol..

